# HIV, STI, and Hepatitis Among People Who Inject Drugs at a Sexual Health Clinic in Melbourne, Australia: 2012 to 2022

**DOI:** 10.1093/ofid/ofaf339

**Published:** 2025-06-13

**Authors:** Tiffany R Phillips, Esha Abraham, Christopher K Fairley, Rayner Kay Jin Tan, Ei T Aung, Jason J Ong, Eric P F Chow

**Affiliations:** Melbourne Sexual Health Centre, Alfred Health, Melbourne, Victoria, Australia; School of Translational Medicine, Faculty of Medicine, Nursing and Health Sciences, Monash University, Melbourne, Victoria, Australia; Melbourne Sexual Health Centre, Alfred Health, Melbourne, Victoria, Australia; School of Translational Medicine, Faculty of Medicine, Nursing and Health Sciences, Monash University, Melbourne, Victoria, Australia; Melbourne Sexual Health Centre, Alfred Health, Melbourne, Victoria, Australia; School of Translational Medicine, Faculty of Medicine, Nursing and Health Sciences, Monash University, Melbourne, Victoria, Australia; Saw Swee Hock School of Public Health, National University of Singapore and National University Health System, Singapore, Singapore; Melbourne Sexual Health Centre, Alfred Health, Melbourne, Victoria, Australia; School of Translational Medicine, Faculty of Medicine, Nursing and Health Sciences, Monash University, Melbourne, Victoria, Australia; Melbourne Sexual Health Centre, Alfred Health, Melbourne, Victoria, Australia; School of Translational Medicine, Faculty of Medicine, Nursing and Health Sciences, Monash University, Melbourne, Victoria, Australia; Melbourne Sexual Health Centre, Alfred Health, Melbourne, Victoria, Australia; School of Translational Medicine, Faculty of Medicine, Nursing and Health Sciences, Monash University, Melbourne, Victoria, Australia; Centre for Epidemiology and Biostatistics, Melbourne School of Population and Global Health, The University of Melbourne, Melbourne, Victoria, Australia

**Keywords:** hepatitis B virus (HBV), hepatitis C virus (HCV), sexually active, sexually transmitted infections (STIs), syphilis trends

## Abstract

**Background:**

This study aimed to examine the sexual practices and sexually transmitted infection (STI) positivity among people who inject drugs (PWID).

**Methods:**

This was a repeated cross-sectional study analyzing data collected at a sexual health center during 2012–2022. New clients who were aged 18 and older, sexually active, and had injected drugs in the last 12 months were eligible for inclusion. Clients were categorized as men who have sex with women only (MSWO), gay or bisexual men who have sex with men (gbMSM), or women. We calculated the 2-year positivity of HIV, STIs, hepatitis B virus (HBV), and hepatitis C virus (HCV), and temporal analyses were conducted using the chi-square trend test.

**Results:**

A total of 1229 clients (395 MSWO, 457 gbMSM, and 377 women) were included in the study. There was a significant rise in syphilis (*P*_trend_ = .0033); however, no significant changes were observed for other infections. The 2-year positivity for syphilis increased significantly from 0.6% (1/156) in 2012/2013 to 10.0% (13/130) in 2020/2021 (*P*_trend_ = .0033). gbMSM had higher positivity for any infection (29.1%, 133/457) than MSWO (19.8%, 78/395) and women (17.0%, 64/377; *P* < .001). Positivity of new HIV was 2.6% (95% CI, 1.6%–3.8%; 22/861), infectious syphilis was 6.8% (95% CI, 5.2%–8.7%; 59/866), gonorrhea was 8.6% (95% CI, 6.8%–10.7%; 77/892), chlamydia was 8.7% (95% CI, 7.0%–10.5%; 95/1093), HBV was 0.6% (95% CI, 0.1%–1.6%; 3/545), and HCV antibody was 10.0% (95% CI, 7.4%–13.2%; 45/448). The HCV testing rate was 37.6% (462/1229).

**Conclusions:**

PWID are highly susceptible to STIs and blood-borne infections. Future prevention programs directed at PWID must include increasing the rate of HCV testing and messages related to sexual risk practices to help reduce the burden of disease in PWID.

Injection drug use is uncommon in Australia—only 0.2% of Australians aged 14 years and above reported injecting a drug in the last 12 months in 2022–2023 [1]. People who inject drugs (PWID) frequently experience poorer health outcomes than noninjecting drug users [2, 3] and are vulnerable to blood-borne viruses (BBVs) such as hepatitis B (HBV) and C (HCV), HIV, and other sexually transmitted infections (STIs). A 2017 meta-analysis estimated that 7.8% of PWID globally have HIV, 52.3% are HCV antibody positive, and 9.1% are HBV surface antigen positive (indicating current infection) [4]. Australian Institute of Health and Welfare data suggest that the prevalence of HIV in Australian PWID is much lower; however, the prevalence rose from 1.7% in 2018 to 2.1% in 2022 [1]. This is despite a 51% decrease in new HIV diagnoses from 2011 to 2022 in the general population [5]. Over 4000 needle and syringe programs operate throughout Australia, distributing >50 million sterile needles annually, contributing to this reduced prevalence compared with global figures [6, 7]. Similarly, almost 2 in 5 (39%) PWID had HCV in 2020 [1]. Unsafe injecting practices, such as needle sharing, and condomless sexual practices can increase the risk of transmission of BBV [8, 9]. A cross-sectional study in 2004 of 314 PWID in Melbourne found that condomless sex (65%) and shared injecting equipment (55%) are common and reported a high prevalence of HBV core antibody (previous infection; 33%) and HCV (74%) [10].

There may be overlap between PWID and other priority populations for STI/HIV such as LGBTQ populations. Illicit drug use (including noninjectable drugs) is more common among LGBTQ populations, with almost half (47%) of lesbian, gay, or bisexual people using any illicit drug in 2022–2023, compared with 17% of heterosexual people [1]. The increased use of drugs among LGBTQ populations has been attributed to a combination of minority stress and discrimination as well as social and cultural factors (including higher rates of chemsex, the use of illicit drugs to enhance sexual experiences) [11–13]. However, there is a lack of data on the prevalence of injecting drug use specifically in this population. Recent data on STIs and BBV in Australian PWID are scarce, and few studies have analyzed the relationship between PWID and sexual practices. A better understanding of current rates of STIs and BBVs in this population is needed to develop harm reduction programs. As such, this study aimed to examine the sexual practices and positivity of HIV, STIs, and viral hepatitis among PWID and to determine whether differences exist between men who have sex with women only (MSWO), gay and bisexual men and other men who have sex with men (gbMSM), and female PWID. A secondary aim was to examine the temporal trends of STI positivity among PWID from 2012 to 2021.

## METHODS

### Study Design and Setting

This was a repeated cross-sectional study analyzing retrospectively collected electronic data from 2012 to 2022. We included individuals who presented to the Melbourne Sexual Health Centre (MSHC) in Australia for the first time between 2012 and 2022 who identified as male or female and reported intravenous drug use within the last 12 months. MSHC is a free, publicly funded sexual health clinic based in inner-city Melbourne, providing free HIV/STI testing and treatment.

### Participants

New clients who identified as male or female, were aged ≥18 years and had had a sexual partner in the previous 12 months, and had injected drugs in the last 12 months were eligible for inclusion in this study. Individuals who declined to provide information about their gender or the gender of partners (or did not have any sexual partners) in the last 12 months were excluded from the study as they were unable to be stratified into subgroups for further analysis. Furthermore, a small number of transgender people were excluded from the study as their sexual practices were not collected.

All clients are asked to fill in a computer-assisted self-interview (CASI) questionnaire upon arrival at their first appointment. CASI collected information on their demographic characteristics (eg, age, sex, country of birth, sex worker status), sexual practices (eg, condom use, gender of sexual partners, number of sexual partners) in the last 12 months, any previous STI diagnoses, and injecting drug use in the last 12 months.

Clients were categorized into subgroups based on their sexual practices in the last 12 months. We defined “MSWO” as men who had only had female partners in the last 12 months and “gbMSM” as men who had had at least 1 male partner in the last 12 months. Due to the small sample size, women were not categorized into further subgroups.

### Laboratory Methods

Clients presenting to MSHC were offered testing for HIV, STIs, and viral hepatitis based on their sexual risk profile and clinical concerns. Laboratory results were extracted from the clinic's database. We analyzed the positivity of HIV, *C. trachomatis*, *N. gonorrhoeae,* infectious syphilis, HBV, and HCV. Both infectious syphilis and HIV were diagnosed using serological testing. HBV and HCV were diagnosed from antibody testing. Gonorrhea positivity was based on culture before March 2015 and subsequently by nucleic acid amplification tests (NAATs), as described previously [14]. We defined positivity as the number of positive tests divided by the number of clients tested for that infection. Clients who self-reported a previous HIV or HBV diagnosis at registration were excluded when calculating the positivity.

### Statistical Methods

Descriptive statistics and frequencies were calculated, and 95% CIs of the proportion were calculated using the binomial exact method. The Fisher exact test was used to compare categorical variables (eg, STI positivity, condom use), and the Kruskal-Wallis *H* test was used for continuous variables (eg, age, median number of sexual partners) between MSWO, gbMSM, and women. Pairwise comparisons using the Mann-Whitney *U* test were conducted when there was a significant difference in Kruskal-Wallis *H* test (*P* < .05). When calculating the *P* value, we excluded those who did not answer “yes” or “no” for categorical variables (ie, those who declined to answer) in order to only analyze dichotomous data; however, these data were included in the table for the sake of completeness. We calculated the 2-year positivity of HIV, HBV, HCV, and other STIs by pooling test results over 2-year intervals and calculating the proportion positive. This approach was chosen to increase stability in estimates for data visualization. Temporal analyses were conducted using the Cochran-Armitage test for trend. Where positivity by subgroup and year is presented, trend testing was conducted using annual data to preserve statistical power by maximizing the number of time points.

All statistical analyses were conducted using Stata (version 17, College Station, TX, USA). This manuscript is reported following the Strengthening the Reporting of Observational Studies in Epidemiology (STROBE) guidelines. Ethics approval was obtained from the Alfred Hospital Ethics Committee (346/22).

## RESULTS

**Figure 1.**
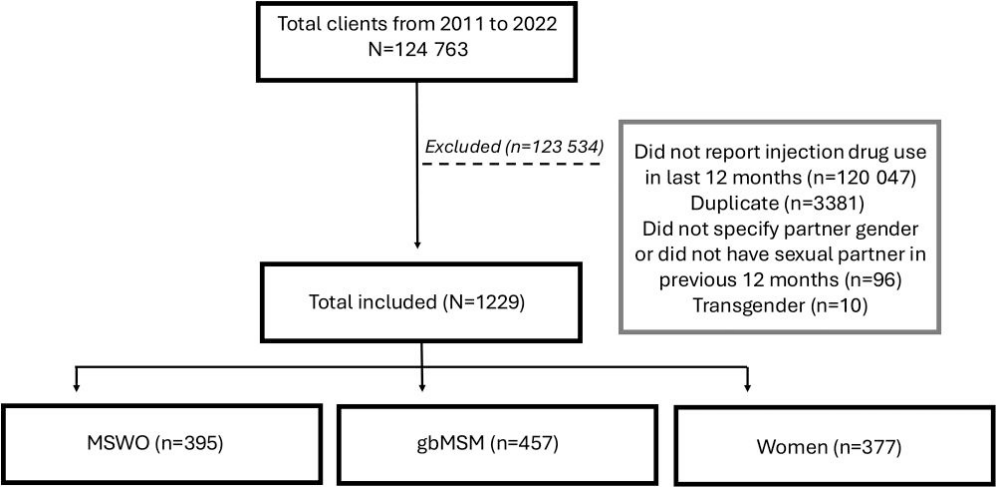
Flowchart for participant inclusion and exclusion. Abbreviations: gbMSM, gay or bisexual men who have sex with men; MSWO, men who have sex with women only.

There was a total of 124 763 clients who first attended MSHC from January 2012 to December 2022 (Figure 1). We excluded a total of 123 534 clients because they did not report injecting drug use in the last 12 months (n = 120 047), were duplicate consultations from the same day (n = 3381), did not specify the gender of their sexual partners in the last 12 months or did not have sexual partners in the last 12 months (n = 96), or identified as transgender (n = 10). The remaining 1229 clients were included in the final analysis, with 395 (32.1%) MWSO, 457 (37.2%) gbMSM, and 377 (30.7%) women. While women were not further subgrouped, the majority had had sex with men only in the previous 12 months (295/377, 78.3%), while 72 (19.1%) had had sex with both men and women and 10 (2.7%) had had sex with only women.

### Demographic Characteristics

Overall, the number of clients who injected drugs presenting to MSHC remained similar between 2012 and 2022. Similarly, the proportion of clients within each subgroup did not change over time (MWSO, *P*_trend_ = .069; gbMSM, *P*_trend_ = .372; women, *P*_trend_ = .385) (Table 1).

**Table 1.** Number of New Presentations of PWID to MSHC Each Year, Stratified by Subgroup

Year	MSWO, No. (%)	gbMSM, No. (%)	Women, No. (%)	Total
2012	40 (35.7)	36 (32.1)	36 (32.1)	112
2013	43 (37.0)	33 (28.4)	40 (34.5)	116
2014	36 (31.0)	43 (37.1)	37 (31.9)	116
2015	42 (37.2)	40 (35.4)	31 (27.4)	113
2016	36 (32.7)	44 (40.0)	30 (27.3)	110
2017	30 (28.3)	45 (42.5)	31 (29.2)	106
2018	36 (24.8)	57 (39.3)	52 (35.9)	145
2019	42 (30.2)	49 (35.3)	48 (34.5)	139
2020	30 (35.3)	33 (38.8)	22 (25.9)	85
2021	28 (29.8)	35 (37.2)	31 (33.0)	94
2022	32 (34.4)	42 (45.2)	19 (20.4)	93
Total	395 (32.1)	457 (37.2)	377 (30.6)	1229
*P*_trend_	.069	.372	.385	

Abbreviations: gbMSM, gay or bisexual men who have sex with men; MSHC, Melbourne Sexual Health Centre; MSWO, men who have sex with women only; PWID, people who inject drugs.

Overall, the median age (interquartile range [IQR]) was 30 (25–38) years, and gbMSM had the highest median (IQR) age (33 [27–41] years), followed by MSWO (30 [25–35] years) and women (26 [22–34] years; *P* < .001) (Table 2). A total of 44 clients (3.6%) identified as Aboriginal or Torres Strait Islander, mostly women (n = 23, 52.2%), followed by gbMSM (n = 13, 29.5%) and MSWO (n = 8, 18.2%; *P* = .005). There were 148 clients (12.7%) who identified as a current sex worker, and the majority (n = 124, 83.7%) were women.

**Table 2.** Comparison of Demographic Characteristics Among MSWO, gbMSM, and Women who Inject Drugs Attending the Melbourne Sexual Health Centre From 2012 to 2021

	MSWO (n = 395)	gbMSM (n = 457)	Women (n = 377)	Total (n = 1229)	*P* Value (MSWO, gbMSM, and Women)	*P* Value (MSWO and gbMSM)	*P* Value (MSWO and Women)	*P* Value (gbMSM and Women)
Median age (IQR), y	30 (25–35)	33 (27–41)	26 (22–34)	30 (25–38)	<.001	<.001	.003	<.001
Country of birth, No. (%)					.052^a^			
Australia	221 (56.0)	286 (62.6)	189 (50.1)	696 (56.6)				
Overseas	149 (37.7)	156 (34.1)	147 (39.0)	452 (36.8)				
No information given	25 (6.3)	15 (3.3)	41 (10.9)	81 (6.6)				
Aboriginal and Torres Strait Islander					.001	.516	.003	.015
No	337 (85.3)	401 (87.8)	294 (78.0)	1032 (84.0)				
Yes	8 (2.0)	13 (2.8)	23 (6.1)	44 (3.6)				
No information given	50 (12.7)	43 (9.4)	60 (15.9)	153 (12.5)				
Current sex worker, No. (%)								
No	387 (98.0)	429 (93.9)	272 (72.2)	1088 (88.5)				
Yes	6 (1.5)	19 (4.2)	97 (25.7)	122 (9.9)	<.001	.024	<.001	<.001
No information given	2 (0.5)	9 (2.0)	8 (2.1)	19 (1.6)				
Median No. of female sexual partners in the previous 12 mo (IQR)	5 (2–9)	3 (1–8)	1 (1–2)	3 (1–7)	<.001	.047	<.001	<.001
Median No. of male sexual partners in the previous 12 mo (IQR)	NA	10 (3–21)	3 (2–6)	5 (2–12)	<.001	-	-	<.001
Median No. of total sexual partners in the previous 12 mo (IQR)	5 (2–9)	11 (5–25)	4 (2–6)	5 (2–11)	<.001	<.001	<.001	<.001
Condom use with female partners in the last 12 mo, No. (%)					.246			
Always	25 (6.3)	9 (9.9)	NA					
Not always	354 (89.6)	76 (83.5)	NA					
No information given	16 (4.1)	6 (6.6)	NA					
Condom use with male partners in the last 12 mo, No. (%)					.897			
Always	NA	37 (8.1)	29 (7.9)					
Not always	NA	386 (84.5)	321 (87.5)					
No information given	NA	34 (7.4)	17 (4.6)					
Previous STI diagnosis, No. (%)					<.001	<.001	.466	<.001
No	217 (54.9)	131 (28.7)	185 (49.1)					
Yes	125 (31.7)	271 (59.3)	120 (31.8)					
No information given	53 (13.4)	55 (12.0)	72 (19.1)					

Abbreviations: gbMSM, gay or bisexual men who have sex with men; IQR, interquartile range; MSWO, men who have sex with women only; STI, sextually transmitted infection.

^a^No subgroup comparisons were made if the overall *P* value was insignificant.

MSWO had the highest median (IQR) number of female partners in the last 12 months (5 [2–9]), followed by gbMSM (3 [1–8]) and then women (1 [1–2]; *P* < .001). gbMSM had a higher median (IQR) number of male partners in the last 12 months than women (11 [5–25] vs 3 [2–6]; *P* < .001). The proportion of gbMSM who reported any previous STI infection (59.3%) was higher than MSWO (31.7%) and women (31.8%; *P* < .001).

There was no significant difference in the proportion of MSWO and gbMSM who always used condoms with female partners in the last 12 months (6.3% vs 9.9%; *P* = .246). Similarly, the proportion of gbMSM and women who always used condoms with male partners in the last 12 months did not significantly differ (8.1% vs 7.9%; *P* = .897).

There were 71 (5.8%) clients who indicated that they had HIV on CASI (3/395, 0.8%, MSWO; 66/457, 14.4%, gbMSM; and 2/377, 0.5%, women) and 56 (4.5%) who had previously tested positive for HCV (16/395, 4.1%, MSWO; 32/457, 7.0%, gbMSM; and 8/377, 2.1%, women). Among all participants, 462 (37.6%) were tested for HCV (including 14 who previously tested positive for HCV). There were 14 (1.1%) clients who indicated they had previously tested positive for HBV (3/395, 0.8%, MSWO; 10/457, 2.2%, gbMSM; 1/377, 0.3%, women).

Overall, gbMSM had a higher positivity for any infection (29.1%, 133/457) than MSWO (19.8%, 78/395) and women (17.0%, 64/377; *P* < .001). HIV positivity was highest among gbMSM (5.7%, 18/317), followed by MSWO (1.4%, 4/271), then women (0.0%, 0/269; *P* < .001). gbMSM were more likely to test positive for infectious syphilis (11.6%, 38/328) than MSWO (3.3%, 9/273) and women (4.5%, 12/265; *P* < .001). Gonorrhea positivity was significantly higher in both MSWO (9.8%, 19/194) and gbMSM (11.7%, 47/401) than in women (3.7%, 11/297; *P* < .001). There were no differences in chlamydia, HBV, or HCV positivity between the 3 groups (Table 3).

**Table 3.** Comparison of HIV, STIs, HBV, and HCV Among MSWO, gbMSM, and Women who Inject Drugs Presenting to the Melbourne Sexual Health Centre From 2012 to 2022

	MSWO (n = 395)			gbMSM (n = 457)			Women (n = 377)						
	Total Tests, No.	Positive Tests, No.	% Positive Cases (95% CI)	Total Tests, No.	Positive Tests, No.	% Positive Cases (95% CI)	Total Tests, No.	Positive Tests, No.	% Positive Cases (95% CI)	*P* Value (MSWO, gbMSM, and Women)	*P* Value (MSWO and gbMSM)	*P* Value (MSWO and Women)	*P* Value (gbMSM and Women)
Chlamydia	352	33	9.4 (6.5–12.9)	404	30	7.4 (5.0–10.4)	337	32	9.5 (6.5–13.1)	.513	-	-	-
Gonorrhea	194	19	9.8 (6.0–14.9)	401	47	11.7 (8.7–15.3)	297	11	3.7 (1.8–6.5)	.001	.290	.006	<.001
Syphilis	273	9	3.3 (1.5–6.2)	328	38	11.6 (8.3–15.5)	265	12	4.5 (2.3–7.7)	<.001	<.001	.510	.003
HBV^a^	147	2	1.4 (0.2–4.8)	257	1	0.4 (0.0–2.1)	141	0	0.0 (0.0–0.0)	.344	-	-	-
HCV	147	19	12.9 (8.0–19.4)	201	19	9.5 (5.8–14.4–11.2)	114	19	16.7 (12.5–29.5)	.168	-	-	-
HIV^b^	275	4	1.5 (0.4–3.7)	317	18	5.7 (3.3–8.8)	269	0	0.0 (0.0–0.0)	<.001	.008	.124	<.001
Any infection	-	78		-	133		-	64		<.001	<.002	.353	<.001

Abbreviations: CASI, computer-assisted self-interview; gbMSM, gay or bisexual men who have sex with men; HBV, hepatitis B virus; HCV, hepatitis C virus; MSWO, men who have sex with women only; STI, sexually transmitted infection.

^a^Excluding those with previous HBV infection.

^b^Excludes 9 participants who indicated on CASI that they had previously been diagnosed with HIV.

There were 36 clients who tested positive for >1 infection (2.9%, 36/1229), of whom 21 were gbMSM (4.6%, 21/457), 8 were MSWO (2.3%, 8/395), and 7 were women (1.9%, 7/377). The most common coinfections were chlamydia and gonorrhea (7/36, 19.4%), syphilis and HCV (6/36, 16.7%), and gonorrhea and HCV (4/36, 11.1%).

**Figure 2.**
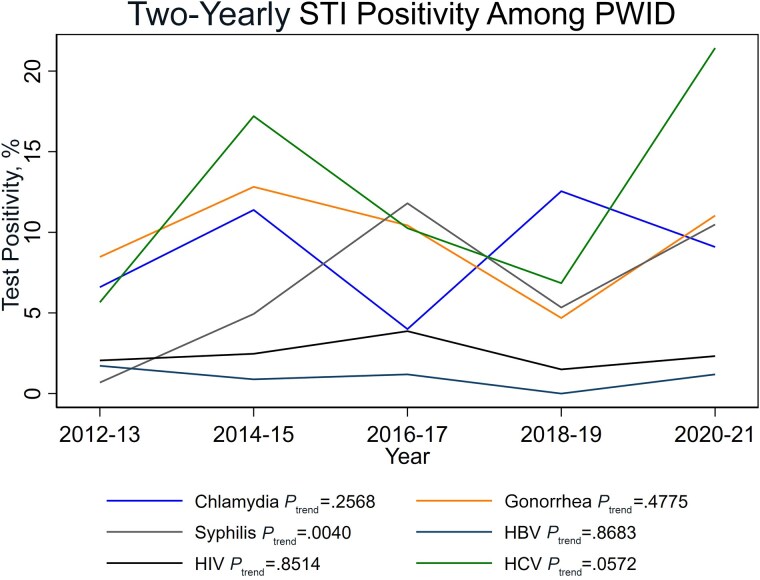
The 2-year positivity of HIV, STIs, HBV, and HCV among people who inject drugs presenting to the Melbourne Sexual Health Centre from 2012 to 2021. Abbreviations: HBV, hepatitis B virus; HCV, hepatitis C virus; PWID, people who inject drugs; STI, sexually transmitted infection.

Overall, the positivity of new HIV was 2.6% (95% CI, 1.6–3.8; 22/861), infectious syphilis was 6.8% (95% CI, 5.2–8.7; 59/866), gonorrhea was 8.6% (95% CI, 6.8–10.7; 77/892), chlamydia was 8.7% (95% CI, 7.0–10.5; 95/1093), HBV was 0.6% (95% CI, 0.1–1.6; 3/545), and HCV was 12.3% (95% CI, 9.5–15.7; 57/462). The 2-year positivity of syphilis increased significantly from 0.6% (1/156) in 2012/2013 to 10.0% (13/130) in 2020/2021 (*P*_trend_ = .0040). The positivity of other infections did not change significantly during this period (Figure 2). When examining infection positivity by subgroup, a significant trend was observed in HCV positivity among MSWO from 2012 to 2022 (*P*_trend_ = .0399), indicating a general increase over time (Supplementary Table 1). No other significant temporal trends were observed for any infection within the other subgroups.

## DISCUSSION

This study of 1229 sexually active PWID presenting to a sexual health clinic in Melbourne between 2012 and 2022 reported a high positivity of HCV (12.3%), gonorrhea (8.6%), and chlamydia (8.7%). The positivity for infectious syphilis was 6.8%. Among gbMSM who were PWID, the positivity reached 8.3% (38/457). These findings are broadly in line with national syphilis incidence estimates in gay and bisexual men attending sexual health clinics, which ranged from 5.5 to 8.1 new infections per 100 person-years in 2021 [15]. Notably, the proportion of PWID testing positive for infectious syphilis increased significantly over the study period (*P*_trend_ = .003), underscoring the growing burden of syphilis in this population and reinforcing the need for intensified screening and prevention efforts.

PWID who are also gbMSM had the highest positivity of any BBV compared with MSWO and women. The high BBV positivity may be explained by a high proportion of condomless sex, as 84.5% of gbMSM did not always use condoms with male partners in the last 12 months.

Almost 90% of PWID in our study reported condomless sex in the last 12 months, which may increase the risk of acquiring and transmitting STIs and BBV [16]. In comparison, a previous study of heterosexuals attending the same clinic during the study period (2019) reported that 50.1% (358/677) had engaged in condomless vaginal sex in the previous 3 months [17]. Similarly, a study reporting condom use rates among sexually active gay and bisexual men in Australia during the study period (2014–2018) found that up to 50.4% (3135/6226) of participants had engaged in condomless anal sex in the previous 6 months (though this varied according to age and whether this was with regular sexual partners vs casual sexual partners) [18]. While it is not possible to directly compare the condom use rates in this current study with those from other studies given that the time frame of condom use reported varies (ie, in the last 12 months vs 3 months vs 6 months), it is still evident that a high proportion of our population of PWID had engaged in condomless sex in the previous year.

A high proportion of condomless sex among PWID has also been reported in other studies and settings. A US study of 10 348 PWID revealed that 67% of participants (including gbMSM) without HIV had had condomless vaginal sex in the last 12 months [19]. Similarly, a study among young PWID in New York City reported that 84.9% had had condomless sex [16]. Other risk factors identified in the previous literature include a greater number of recent sexual partners and having sex in exchange for money or drugs [20–22]. While the use of injecting drugs may influence sexual risk perception, it is likely that social and economic factors also play a role as PWID may have difficulty accessing medical care and condoms. Further research among this population is warranted to inform harm reduction programs.

There was a concerningly low rate (37.6%) of HCV testing among our participants given that our sample was from sexually active first-time attendees to the clinic who indicated that they had injected drugs in the previous 12 months. The Australian STI Management Guidelines indicate that an annual HCV test is warranted among PWID as even those who have been treated and cured of their HCV in the past may be at risk of re-infection if they continue to inject drugs [23]. Our low testing rate is consistent with a modeling study that showed that to reach the World Health Organization targets of 80% of people with hepatitis C being treated and incidence being reduced to 80% of the 2015 level by 2030, we have to increase testing rates by 50%, particularly among young PWID [24].

This study has several limitations. First, our study was conducted at a single public sexual health service in metropolitan Melbourne and thus our results are biased toward those engaged in sexual health services (who may have symptoms of an STI at presentation). Thus, our findings may not be generalizable to the wider population of sexually active PWID in Australia and other settings. Second, we stratified individuals into different subgroups based on their self-reported sexual practices, and this may not correlate with their sexual identity and orientation [25, 26]. Third, we did not collect data on types of substances injected or whether the drug use was in a sexualized context (ie, for use before sex, also known as “chemsex”), which may have implications for STI transmission. Fourth, our clinic changed the diagnosis methods from culture to NAAT for gonorrhea in 2015. Furthermore, we expanded gonorrhea screening to women and heterosexual men in our clinic in August 2017 [27]. These changes may have led to an increase in gonorrhea cases, although the positivity of gonorrhea did not change over time. Finally, we were unable to stratify women based on their sexual practices due to the small sample size. A past Melbourne-based study found that STI positivity and sexual practices differed among women who have sex with women only, women who have sex with men and women, and women who have sex with men only [28], so further research into women who inject drugs is warranted.

## CONCLUSIONS

PWID are at high risk for BBVs and STIs, and gbMSM are at a greater risk than MSWO and women. Rates of sexual risk behaviors, such as multiple partners and condomless sex, were high among the PWID in our study. Our study highlights the need for further research into the relationship between PWID and sexual risk behaviors. Future programs to improve the health and well-being of PWID must focus on increasing the rate of testing for HCV and include messages related to sexual risk practices to help reduce the burden of disease in this population.

## Supplementary Material

ofaf339_Supplementary_Data

## Supplementary Data

Supplementary materials are available at *Open Forum Infectious Diseases* online. Consisting of data provided by the authors to benefit the reader, the posted materials are not copyedited and are the sole responsibility of the authors, so questions or comments should be addressed to the corresponding author.
