# Supplementary material for: HIV, STI, and Hepatitis Among People Who Inject Drugs at a Sexual Health Clinic in Melbourne, Australia: 2012 to 2022
Source: Open Forum Infect Dis. 2025 Jun 13;12(7):ofaf339. doi: 10.1093/ofid/ofaf339 (PMC12207969; doi:10.1093/ofid/ofaf339)
Supplement: ofaf339_Supplementary_Data [file ofaf339_supplementary_data.docx]

Appendix Table 1: Positivity of chlamydia, gonorrhoea, syphilis, HBV, HCV, and HIV among MSWO, gbMSM and women who inject drugs presenting to the Melbourne Sexual Health Centre from 2012-2022

|  | 2012 (n/N; %) | 2013 (n/N; %) | 2014 (n/N; %) | 2015 (n/N; %) | 2016 (n/N; %) | 2017 (n/N; %) | 2018 (n/N; %) | 2019 (n/N; %) | 2020 (n/N; %) | 2021 (n/N; %) | 2022 (n/N; %) | Ptrend* |
| --- | --- | --- | --- | --- | --- | --- | --- | --- | --- | --- | --- | --- |
| Chlamydia |  |  |  |  |  |  |  |  |  |  |  |  |
| MSWO | 5/36; 13.9 | 3/39; 7.7 | 2/32; 6.2 | 6/40; 15.0 | 1/32; 3.1 | 0/28; 0.0 | 4/29; 13.8 | 5/38; 13.2 | 1/24; 4.2 | 2/25; 8.0 | 4/29; 13.8 | 0.9797 |
| gbMSM | 1/27; 3.7 | 1/28; 3.6 | 4/36; 11.1 | 5/34; 14.7 | 2/40; 5.0 | 4/43; 9.3 | 3/51; 5.9 | 3/46; 6.5 | 4/29; 13.8 | 2/32; 6.2 | 1/38; 2.6 | 0.5975 |
| Women | 3/33; 9.1 | 0/34; 0.0 | 2/31; 6.5 | 4/29; 13.8 | 0/28; 0.0 | 1/29; 3.4 | 10/49; 20.4 | 7/42; 16.7 | 2/18; 11.1 | 3/26; 11.5 | 0/18; 0.0 | 0.1635 |
| Gonorrhoea |  |  |  |  |  |  |  |  |  |  |  |  |
| MSWO | 2/4; 50.0 | 2/5; 40.0 | 0/2; 0.0 | 1/10; 10.0 | 0/12; 0.0 | 1/17; 5.9 | 1/29; 3.4 | 0/38; 0.0 | 5/24; 20.8 | 4/25; 16.0 | 3/28; 10.7 | 0.5392 |
| gbMSM | 2/25; 8.0 | 4/27; 14.8 | 4/35; 11.4 | 7/34; 20.6 | 8/41; 19.5 | 3/43; 7.0 | 7/51; 13.7 | 3/46; 6.5 | 4/29; 13.8 | 3/32; 9.4 | 2/38; 5.3 | 0.1907 |
| Women | 0/27; 0.0 | 0/30; 0.0 | 1/20; 5.0 | 2/16; 12.5 | 2/24; 8.3 | 3/26; 11.5 | 1/49; 2.0 | 0/43; 0.0 | 0/18; 0.0 | 1/26; 3.8 | 1/18; 5.6 | 0.9546 |
| Syphilis |  |  |  |  |  |  |  |  |  |  |  |  |
| MSWO | 0/26; 0.0 | 0/27; 0.0 | 0/21; 0.0 | 0/35; 0.0 | 2/26; 7.7 | 2/23; 8.7 | 0/23; 0.0 | 2/30; 6.7 | 1/18; 5.6 | 1/22; 4.5 | 1/22; 4.5 | 0.0797 |
| gbMSM | 1/19; 5.3 | 0/21; 0.0 | 3/33; 9.1 | 3/29; 10.3 | 5/34; 14.7 | 7/28; 25.0 | 4/44; 9.1 | 2/38; 5.3 | 5/24; 20.8 | 3/27; 11.1 | 5/31; 16.1 | 0.1413 |
| Women | 0/23; 0.0 | 0/30; 0.0 | 0/21; 0.0 | 2/23; 8.7 | 2/24; 8.3 | 1/26; 3.8 | 1/38; 2.6 | 2/33; 6.1 | 2/12; 16.7 | 1/21; 4.8 | 1/14; 7.1 | 0.0821 |
| HBV |  |  |  |  |  |  |  |  |  |  |  |  |
| MSWO | 0/23; 0.0 | 1/20; 5.0 | 0/10; 0.0 | 0/21; 0.0 | 1/11; 9.1 | 0/8; 0.0 | 0/8; 0.0 | 0/6; 0.0 | 0/10; 0.0 | 0/15; 0.0 | 0/15; 0.0 | 0.4420 |
| gbMSM | 0/16; 0.0 | 0/15; 0.0 | 0/26; 0.0 | 0/25; 0.0 | 0/22; 0.0 | 0/23; 0.0 | 0/31; 0.0 | 0/35; 0.0 | 1/17; 5.9 | 0/22; 0.0 | 0/25; 0.0 | 0.3618 |
| Women | 0/17; 0.0 | 0/23; 0.0 | 0/16; 0.0 | 0/12; 0.0 | 0/12; 0.0 | 0/8; 0.0 | 0/12; 0.0 | 0/11; 0.0 | 0/6; 0.0 | 0/14; 0.0 | 0/10; 0.0 | -- |
| HCV |  |  |  |  |  |  |  |  |  |  |  |  |
| MSWO | 1/22; 4.5 | 0/19; 0.0 | 0/7; 0.0 | 3/23; 13.0 | 5/12; 41.7 | 1/10; 10.0 | 2/8; 25.0 | 0/7; 0.0 | 0/12; 0.0 | 4/14; 28.6 | 3/13; 23.1 | 0.0399 |
| gbMSM | 1/16; 6.2 | 1/17; 5.9 | 4/24; 16.7 | 4/20; 20.0 | 0/19; 0.0 | 0/16; 0.0 | 0/18; 0.0 | 1/19; 5.3 | 1/11; 9.1 | 5 /19; 26.3 | 2/22; 9.1 | 0.6288 |
| Women | 2/16; 12.5 | 1/16; 6.2 | 3/11; 27.3 | 2/8; 25.0 | 2/11; 18.2 | 0/10; 0.0 | 1/10; 10.0 | 1/11; 9.1 | 1/4; 25.0 | 4/10; 40.0 | 2/7; 28.6 | 0.1762 |
| HIV |  |  |  |  |  |  |  |  |  |  |  |  |
| MSWO | 0/27; 0.0 | 0/28; 0.0 | 0/21; 0.0 | 0/36; 0.0 | 1/23; 4.3 | 2/22; 9.1 | 0/24; 0.0 | 0/28; 0.0 | 1/20; 5.0 | 0/23; 0.0 | 0/23; 0.0 | 0.6492 |
| gbMSM | 2/19; 10.5 | 1/20; 5.0 | 0/30; 0.0 | 4/32; 12.5 | 1/33; 3.0 | 2/27; 7.4 | 1/41; 2.4 | 2/35; 5.7 | 1/23; 4.3 | 1/26; 3.8 | 3/31; 9.7 | 0.9757 |
| Women | 0/22; 0.0 | 0/30; 0.0 | 0/21; 0.0 | 0/22; 0.0 | 0/25; 0.0 | 0/25; 0.0 | 0/38; 0.0 | 0/34; 0.0 | 0/15; 0.0 | 0/22; 0.0 | 0/15; 0.0 | -- |

Abbreviations: “MSWO”: men who have sex with women only; “gbMSM”: gay, bisexual and other men who have sex with men

*Ptrend calculated using the Cochran-Armitage test for trend on the annual data
